# A Multidisciplinary Comparison of Different Techniques Among Skilled Water Treaders

**DOI:** 10.3389/fphys.2021.719788

**Published:** 2021-08-20

**Authors:** Tina van Duijn, Chris Button, Rich S. W. Masters

**Affiliations:** ^1^School of Physical Education, Sport and Exercise Sciences, University of Otago, Dunedin, New Zealand; ^2^Te Huataki Waiora School of Health, University of Waikato, Hamilton, New Zealand

**Keywords:** water safety, water polo, cognitive load, economy, probe reaction time, physical load, oxygen consumption

## Abstract

In an immersion incident, a person may be required to tread water for extended periods of time in order to survive. Treading water, or maintaining a stable head position above the water surface, can be achieved in several different ways. Determining which treading water techniques are economic (energetically and cognitively) is an important first step in approaching evidence-based water safety instruction. The present study investigated the cognitive and metabolic demands associated with four main techniques for treading water in experienced water treaders. Skilled water treaders (*n*=21) performed four common treading techniques for 3min each: “running” in the water, “flutter kick” with hands sculling, “upright breaststroke,” and “egg-beater.” Self-reported rate of perceived exertion (RPE) and task load index (TLX) score, as well as objective measures of probe reaction time (PRT; i.e., response to auditory cues while treading), oxygen consumption and heart rate were assessed. The “egg-beater” technique and the “upright breaststroke” technique were linked to significantly lower cognitive and energetic demands compared to the other techniques (VO_2_: *p*<0.001 – “Running” *M*=29.02, *SD*=7.40/“Flutter kick” *M*=29.37, *SD*=8.56, “Breaststroke” *M*=23.47, *SD*=7.28, and “Eggbeater” *M*=23.18, *SD*=6.31). This study lays the groundwork for future research that may establish the ideal movement behavior in drowning situations and investigate movement instruction to less experienced treaders.

## Introduction

Treading water is one of the most versatile of physical water competencies and it is a crucial survival skill for drowning prevention (Stallman et al., 2017). It involves maintaining an upright position with the head above the water, which may serve numerous functions, including resting, maintaining visibility, assessing the surrounding environment, waiting for rescue, communication, signaling for help, saving others, and reducing heat loss (Golden and Tipton, 2002). Treading water can require significant cognitive and physical effort (Graham, 1977), which may hasten the onset of hypothermia and exhaustion (Hayward et al., 1975b; Stallman et al., 2017) and lead to errors in decision making, such as whether to swim to safety and distance judgments (Ducharme and Lounsbury, 2007). People tread water in a variety of ways: for example, a person might naturally adopt a “running” type movement, a “flutter kick,” or an “egg-beater” technique (Schnitzler et al., 2015). In order to be able to provide recommendations for effective, evidence-based instruction of this skill, a more detailed investigation of the cognitive and energetic demands of different treading water techniques is required.

According to Sparrow and Newell (1998), “economical movements are those that achieve the task goal with relatively low metabolic energy expenditure for the given task demands” (p. 173). Von Döbeln and Holmér (1974) were among the first to study the energetic demands of treading water. They determined that a linear relationship exists between oxygen consumption and sinking force. Indeed, when body loading was increased with a weighted jacket, each additional kilogram increased VO_2_ by about 1/3L.min^−1^. Hayward et al. (1975a,b) showed that different motor behaviors have large effects upon the cooling rates people experience when submerged in open water. When there was no life jacket available and participants were required to actively tread water, the cooling rate increased by 35% when compared to resting in the heat escape lessening posture (HELP).^1^ More recently, Barwood et al. (2016) showed that leg only exercise (kicking) in contrast to resting while immersed in water increased metabolic demand in terms of elevated oxygen uptake and breathing frequency (by 18 and 15% respectively over 5min). These studies indicate that the behavior or movement pattern that is employed has an influence on energy requirements. From research in swimming, this is well-documented: for example, effects of technique on oxygen uptake have been found in competitive swimming (Holmér, 1972). Capelli et al. (1998) also found clear differences in energetic demands between swimming techniques, with front-crawl the most economic stroke. Interestingly, the relationship of economy with swimming speed differed as a function of technique: economy increased exponentially with speed in crawl and backstroke, whereas in butterfly, economy only increased exponentially at higher speeds. The energetic cost in breaststroke showed a linear relation with speed, potentially due to the high drag forces associated with the leg recovery phase. Zamparo et al. (2008) found that changes in the swimmer’s position in the water greatly influence energy expenditure (e.g., a more streamlined position requires less energy), which may be a significant factor in mediating the effect of technique.

In treading water, technique has also been proposed as a main factor influencing physiological load and energy requirement. Schnitzler et al. (2015) identified eight behavioral types of treading water across a range of skill levels, which they distilled into four distinctive movement patterns: (1) vertical movements of hands and feet – “running” in the water; (2) feet kicking and hands sculling – “flutter kick”; (3) feet sculling synchronously – “upright breaststroke”; and (4) feet sculling asynchronously – “egg-beater” (see Table 1). It has been suggested that differences in the patterns regarding the physical mechanism of generating lift are reflected in energetic economy (see also Sanders, 1999). Some water-treading patterns, such as the “running” pattern, are based on the use of pushing forces applied to the water (i.e., vertical movements), whereas patterns such as the “egg-beater kick” are characterized by lateral, asynchronous movements that generate more continuous lift forces (Schnitzler et al., 2014, 2015). The “egg-beater” technique is generally believed to be the most economic; however, this needs to be directly confirmed in skilled water treaders (Homma and Homma, 2005).

**Table 1:** Treading techniques.

Category in Schnitzler et al. (2015)	Type 1.2	Type 2.1	Type 3.1	Type 4.1
Description	Vertical movements of hands and feet	Feet flutter kicking and hands sculling	Feet and hands sculling synchronously	Feet sculling asynchronously, hands sculling
Short description	Running	Flutter kick	Upright breaststroke	Egg-beater

Another influential factor in predicting economy of movement in water seems to be the quality of movement execution. In competitive swimming, for example, effects on energy cost have been documented for skill level (Toussaint, 1990), propulsion efficiency (i.e., high lift-to-drag ratio), and technique/coordination level (Seifert et al., 2010). Skilled performers are generally better able to optimize the coupling of their intrinsic dynamics with the demands of a task (Seifert et al., 2013). Therefore, the energy requirement of treading water is likely dependent on skill level. Based on findings of lower rate of perceived exertion (RPE), Schnitzler et al. (2014) suggested that skilled water treaders require less energy than unskilled water treaders. Moreover, skilled treaders may choose to perform more complex, but more economic, patterns. Novices show a global tendency to synchronize the limbs rather than to exhibit asynchrony (as documented in rhythmic movements; Oullier et al., 2006), which would lead to movements with a large recovery phase. Furthermore, less skilled water-treaders may rely on the use of pushing forces (as described above), which include a recovery phase in which no lift is created, whereas more skilled treaders may rely on movements that generate lift continuously (and thus more economically; Schnitzler et al., 2014, 2015). Schnitzler and colleagues found that the egg-beater technique was predominantly performed by experts, while patterns 1 and 2 were performed mainly by less experienced swimmers (Schnitzler et al., 2014).

In order to determine which movement forms are most useful in a drowning scenario, it makes sense to investigate the cognitive load of different treading patterns alongside energetic load: In an open water emergency, one has to make complex decisions under time pressure, while treading water at the same time (Golden and Tipton, 2002). Decision making requires the integration of perceptual information with existing knowledge, and therefore places a high demand on cognitive resources (Raab, 2003). Because conscious control of a complex movement also depends on the same, limited, cognitive resources (Maxwell et al., 2003; Baddeley, 2012), the demands that result from multiple task requirements (i.e., decision making and movement execution) are likely to overload the performer and disrupt performance (Poolton et al., 2006). However, the extent to which control of a movement draws from cognitive resources depends on the amount of explicit, declarative processing that is necessary to control it. Poolton et al. (2006) showed that performance of a difficult decision and a concurrent motor task under time pressure can be improved if the cognitive load related to the motor task is reduced; for example, by acquiring the motor task implicitly so that its execution requires little conscious thought. Therefore, being able to perform the motor task of treading water with minimal cognitive effort may be as important as being able to do it with minimal physical effort. Button et al. (2019) contrasted participants who were treading water during changing task and environmental constraints, and identified that “breast-stroke” and “egg-beater” techniques were most stable when factors such as current, clothing, and additional cognitive demands were manipulated. Fatigue has been shown to affect decision making and movement accuracy during treading water (Royal et al., 2006), which shows that there may be a link between cognitive and physical load; however, no research to date has investigated the cognitive requirements associated with performing different treading water techniques.

In summary, the existing literature does not provide enough evidence to form a clear hypothesis as to which technique of treading water may be energetically most economic. Clearly, a direct investigation of the various demands of treading water techniques is required to address this gap in the literature. The present study aimed to investigate the cognitive and metabolic demands associated with the four techniques described above. Rather than investigating all possible movement strategies in a water emergency, this study focused on comparing solely the four most commonly used water treading techniques. To maximize reliability and internal validity, the study was conducted in a controlled, lab-based environment (a swimming flume) where other potentially influential factors (such as temperature, water flow, clothing, and obstacles) were kept identical between participants and trials. In order to fairly compare the energetics of different movement techniques, it is important that each is performed correctly. We therefore tested water treaders that were skilled at each technique, and controlled movement quality with qualitative analyses and ratings of movement execution. To best capture the different challenges associated with performing complex movements, we conducted a *multidisciplinary* analysis of treading water economy (i.e., cognitive, metabolic, cardiac, perceived exertion, and emotional indices).

## Materials and Methods

### Participants

Participants were water polo players (*n*=11, WP playing experience=7.46years, and *SD*=3.75), synchronized swimmers (*n*=1, experience=13years), and competitive swimmers (*n*=9, swimming experience=9years, and *SD*=4.64) who self-identified as water treading experts (12 females, nine males, mean age=24.24years, and SD=6.24). The sample size of 21 was based upon a previous study by Schnitzler et al. (2014). An inclusion criterion was self-reported expertise in all water treading techniques. Exclusion criteria included significant motor impairments, injuries, or existing health conditions (e.g., severe asthma). Participants reported themselves as sufficiently competent to tread water without support for at least 3min. The experiment was approved by the human ethics committee of the participating institution and written informed consent was given prior to commencing the measurements. Participants were on average 169.64cm tall (*SD*=7.51cm), weighed 72.69kg (*SD*=15.58) out of the water and 6.16kg (*SD*=1.19kg) in the water (with head out).

### Materials

Testing occurred in a swimming flume (StreamliNZ, Dunedin, New Zealand). The flume depth was 2m and a treading area of 6m×2m was available. For all trials, the water was still (i.e., the flume not operating). The water temperature was consistently set at 27°C to ensure the data were not influenced by temperature fluctuations (Button et al., 2015; Schnitzler et al., 2018).

Participants’ behaviors were recorded by three HD video cameras (Sony, Tokyo, Japan): one from behind the participant (below water level), and two from the left and right diagonals (above water level). The under-water camera was the principal camera for qualitative analysis of technique, the front-left camera was used for audio-based assessment of probe reaction time (PRT), and the front-right camera was mainly used as back-up in case of equipment malfunction or to help confirm pattern classification. Participants were positioned to face a screen suspended over the pool edge, approximately 0.5m away from the pool side. A ladder as well as bars at the side of the flume channel were available for support while entering or exiting the water, and during breaks.

A snorkel was fitted to participants’ face via a rubber band around their forehead, for measurements of respiratory gases (Button et al., 2019). The snorkel was connected to a respiratory gas sample line and a turbine digital transducer, which measured inspired and expired volume, O_2_ and CO_2_ in the expired air via an automated gas analysis system (CPET, Cosmed, Italy).

Prior to flume testing, each participant’s static buoyancy was determined in a 3m deep tank of water. A plastic chair was attached via a strain gauge (Futek LCM300 250lb., Futek Advanced Sensor Technology Inc., United States, sample frequency: 100Hz) to a mechanized winch, which could be lowered into the tank.

### Procedure

The experiment occurred on one occasion, approximately 2h in duration. Participants first provided basic demographic information as well as height and weight measurements and self-report of swimming and treading water experience. They then undertook the static buoyancy measurement procedure as described in previous reports (Button et al., 2019). Spirometry data (i.e., vital capacity) were collected as part of the buoyancy measurement.

After an experimenter fitted the snorkel and heart rate monitor, each participant was asked to sit quietly for 3min for collection of baseline breath and heart rate data. Participants then carefully entered the flume and rested against a side bar. A note was made of their oxygen consumption during standing rest in the water, for later reference during recovery intervals. Once they felt comfortable breathing through the snorkel, they moved to the center of the flume and performed a warm-up trial unsupported in the water using their individual preferred movement pattern. The warm-up trial was uninstructed, i.e., the participants were asked to tread water in their preferred way. The warm-up trial was always conducted first, to ensure that individual preference for water treading technique would not be influenced by any of the patterns subsequently adopted during the study. Participants then performed one trial of each of the four water-treading techniques (Table 1) for three and a half minutes (210s). This duration was deemed long enough to achieve steady state, without inducing too much fatigue in the subject. Between each trial, participants rested at the side of the flume until their oxygen consumption had returned to within 20% of VO_2_ during standing rest. The order of the remaining techniques was counterbalanced and allocated randomly to participants (i.e., some orders were repeated). For example, underwater videos of a water polo player performing the required technique were presented on the screen for as long as the participant wanted to practice the movements. Participants were told to “keep their head above water,” and to “move like the person on the screen, as exactly as possible.” Participants were allowed to practice each technique until they were certain that they could perform it for 210s continuously, before initiating the test.^2^ During the first 2min of each test, participants performed a concurrent PRT task as described below. The Borg RPE scale was presented to participants on a laminated sheet after 60, 120, and 180s and they were asked to point to their level of perceived exertion. After each trial, participants completed the NASA Task load Index (NASA TLX, Hart and Staveland, 1988, see Measures subsection) while resting at the side of the flume.

### Measures

#### Wet Weight

Given the potential for individual buoyancy to influence energy expenditure (Button et al., 2019), “wet” body weight (i.e., the person’s weight when they are immersed in the water with their head out) was determined for all participants. Participants were asked to sit in a chair suspended above water level with a 20-kg barbell in their lap to stabilize the participant’s position on the chair when submerged. The chair was then winched into the water until the participant’s chin was just above water level. Once the participant and chair were steady, weight measurements (corrected for weight of the barbell) were recorded for 10s, while participants held their breath (to limit movement). To obtain reliable data, this procedure was undertaken three times with rests permitted between attempts–results were averaged over the three trials. The wet weight data were filtered (Butterworth fourth order, cut-off frequency 0.5Hz).^3^

#### Metabolic Load

A snorkel (described above) was connected to a respiratory gas sample line and a turbine digital transducer, which measured inspired and expired volume, O_2_ and CO_2_ in the expired air *via* an automated gas analysis system (QUARK CPET, Cosmed, Rome, Italy). Participants wore a nose clip to ensure no air was expired other than through the mouth. Before and after each test, the volume transducer was calibrated using a manual 3-l syringe (Hans Rudolph, Kansas City, MO, United States), and the gas analyzers were calibrated using room air and a gas mixture of known composition (5% CO_2_, 16% O_2_, and atmospheric Nitrogen). Inspired and expired gas volume and gas concentration signals were continuously sampled (breath-by-breath) from the mask using an analog-to-digital converter (Omnia suite, version 1.0, COSMED, Rome, Italy) and stored for offline analysis in Microsoft Excel. The accepted range for breaths was defined as: VT>0.2l, 2≤RF≤80 breaths/min, 50≤VO_2_≤7,500ml/min, and 0.5≤RQ≤2. Invalid breaths were discarded. Baseline for respiratory, metabolic, and physiological data was calculated as the mean of the last 120s of baseline data. During the experiment, the baseline was used as a reference to ensure sufficient recovery between trials: the next trial was not started until participants’ oxygen consumption was within 20% of their individual baseline. Respiratory, metabolic, and physiological data were averaged over the last 30s of each trial. It was presumed that a steady-state in oxygen consumption would have been reached after 180s, as shown in previous studies comparing the four swim strokes (Capelli et al., 1998) and in water treading (Von Döbeln and Holmér, 1974). As producing a vocal noise through the snorkel (as required during the PRT) introduces artifacts in the measurements, respiratory data were not analyzed for the first 2min of the trial, while the PRT was performed. For analysis, oxygen consumption (originally given in ml/min) was standardized by wet weight (measured in kg) to achieve *relative VO_2_* (ml/kg/min), as VO_2_ and wet weight were strongly correlated (i.e., Pearson *r*=0.419, *p*<0.001 in the present sample).

#### Heart Rate

Heart rate was continuously recorded from the heart’s electrical activity *via* a chest strap (H10; Polar Electro Inc., Kempele, Finland) and digitized into Omnia using and ANT+ port.

#### Perceived Exertion

Borg’s RPE scale was used to assess perceived exertion (Borg, 1990). The scale ranges from 6 (no exertion at all) to 20 (maximal exertion).

#### Probe Reaction Time

A PRT task provided an objective measure of cognitive load of each treading technique. Reaction time in the PRT task has been shown to be reflective of cognitive demand of a concurrently performed motor task (Brisswalter et al., 1995; Wulf et al., 2001). A visual signal (black, large dot) was presented on the screen at random intervals, accompanied by a beep, upon which the participant was asked to respond with a vocal noise (“yes” or similar) through the snorkel as quickly as possible. Pilot tests showed that participants were able to respond with a vocally produced noise without disrupting their breathing through the snorkel. The stimulus was presented 11 times during 120s, the first measurement was discarded as participants were familiarizing with the technique.

#### Perceived Task Load

Self-perceived effort was assessed using a NASA-Task Load Index (NASA TLX, Hart and Staveland, 1988), at the conclusion of each trial. The NASA TLX is a weighted rating scale that assesses six aspects of task load, including the mental, physical, and temporal demands, as well as effort, frustration, and perceived performance. Each scale was presented as a 12-cm line with a title (e.g., MENTAL EFFORT) and bipolar descriptors at each end (e.g., HIGH/LOW). Numerical values were not displayed, but values ranging from 1 to 100 were assigned to scale positions during data analysis.

#### Ratings of Movement Pattern and Quality

In order to check whether participants performed the instructed techniques correctly, two analysts were trained to perform qualitative analysis of treading water according to the method developed by Schnitzler et al. (2014). Both analysts independently identified the coordination pattern in a random sample of the data while blinded to the technique that was instructed. Raters also rated the quality of movement execution on a scale ranging from 1 (low/novice quality) to 10 (high/expert quality), which served as a further indicator of execution quality and is reported in the descriptives table (Table 2). Pearson correlation between raters were calculated for all variables, in *n*=50 cases, i.e., 10 participants×5 techniques. Ratings of movement quality showed acceptable reliability (*r*=0.602, *p*<0.001). Technique ratings showed a 98% agreement between raters.

**Table 2:** Preferred technique and quality of movement execution.

	“Running”	“Flutter kick”	“Upright breaststroke”	“Egg-beater”
Preferred technique during warm-up	0 (0%)	3 (14.3%)	3 (14.3%)	15 (71.4%)
% correct assigned technique during trials	19 (90.5%)	19 (90.5%)	18 (85.7%)	18 (85.7%)
Movement quality *M*(*SD*)	8.95 (0.92)	9.24 (0.83)	9.10 (0.94)	9.52 (1.08)

### Statistical Analysis

A Shapiro-Wilk test showed no departure from normality in PRT [*W*(48)=0.98, *p*=0.50], TLX [*W*(48)=0.97, *p*=0.20], RPE [*W*(48)=0.97, *p*=0.24], and VO_2_ [*W*(48)=0.97, *p*=0.22], but a significant departure from normality in HR [*W*(48)=0.93, *p*=0.005]. Hence, for each variable (i.e., HR, VO_2_, RPE, NASA TLX, and PRT), a separate repeated-measures ANOVA was conducted to compare the four treading techniques (running, flutter kick, upright breast-stroke, and egg-beater). ANOVA has been reported to be robust to violations of the normality assumption (Rasch and Guiard, 2004). Greenhouse-Geisser correction was used in cases where data were non-spherical. *Post-hoc* comparisons were conducted with a Bonferroni correction. The threshold for statistical significance was set to *p*=0.05.

## Results

### Preferred Technique and Quality

Around 71.4% (*N*=15) of participants demonstrated an “egg-beater” movement during the uninstructed warm-up when using their preferred technique (see Table 2). Compliance with instructed techniques during the subsequent instructed trials was very high, with between 85.7 and 90.5% correct execution. Ratings of quality of movement execution ranged from 8.9 to 9.5 out of 10.

### Effect of Treading Technique on Economy

**Figure 1:**
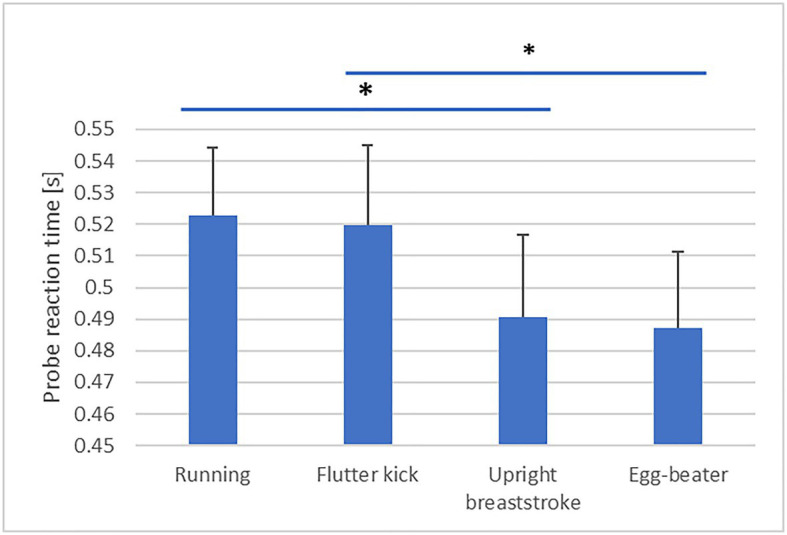
Probe Reaction Time (PRT) during four treading water techniques in skilled treaders – means and SEs. ^*^Denotes significant difference between techniques (*p*_corrected_<0.05).

**Figure 2:**
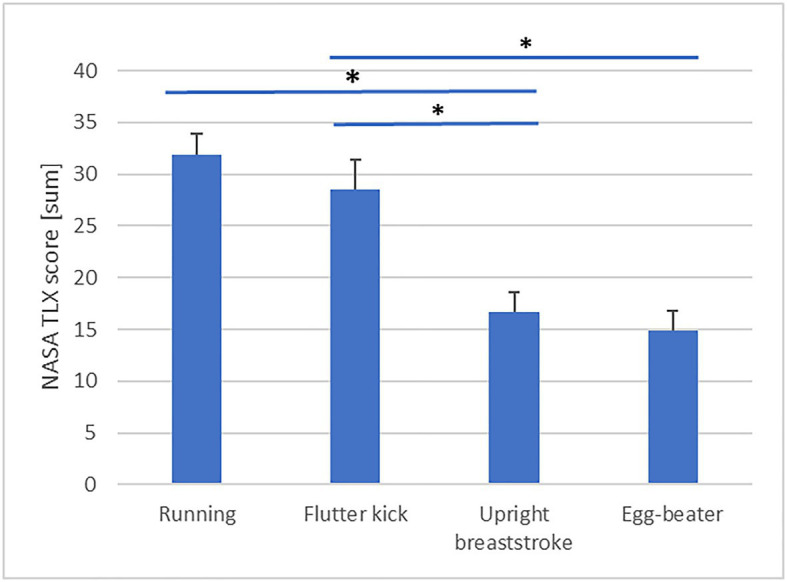
NASA Task load Index (NASA TLX) scores during four treading water techniques in skilled treaders – means and SEs. ^*^Denotes significant difference between techniques (*p*_corrected_<0.05).

**Figure 3:**
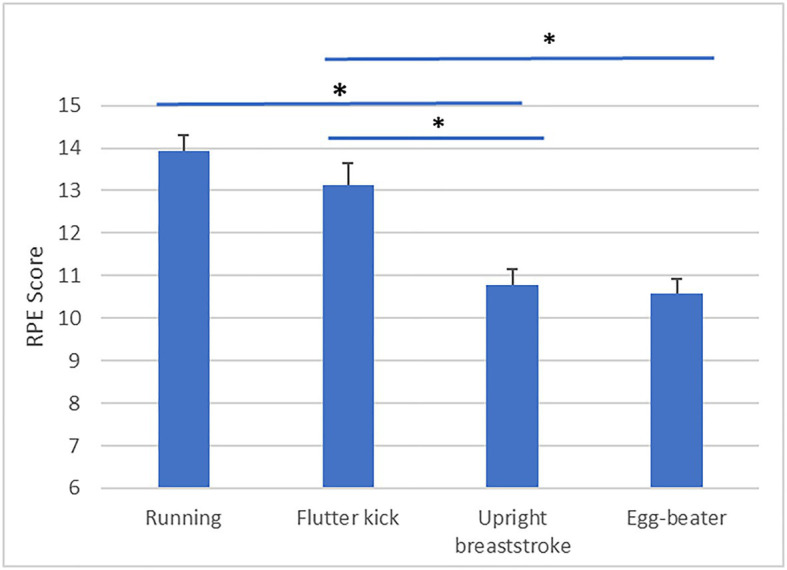
Rate of Perceived Exertion (RPE) during four treading water techniques in experts – means and SEs. ^*^Denotes significant difference between techniques (*p*_corrected_<0.05).

**Figure 4:**
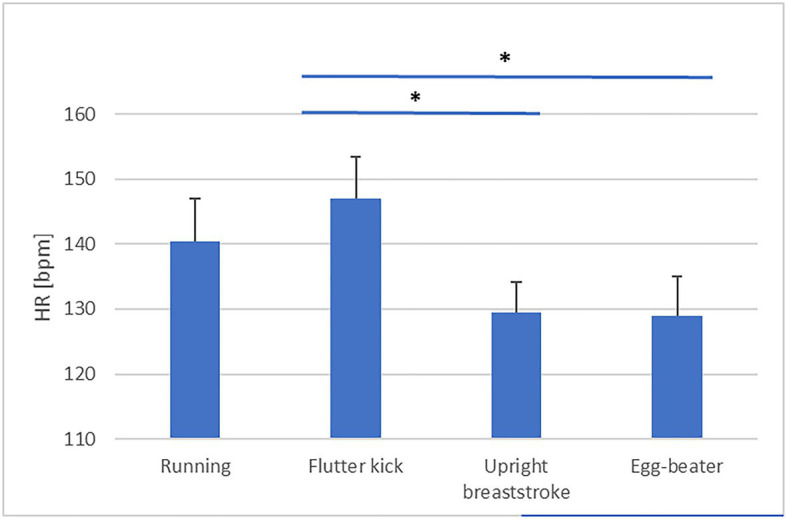
Heart Rate (HR) during four treading water techniques in skilled treaders – means and SEs. ^*^Denotes significant difference between techniques (*p*_corrected_<0.05).

**Figure 5:**
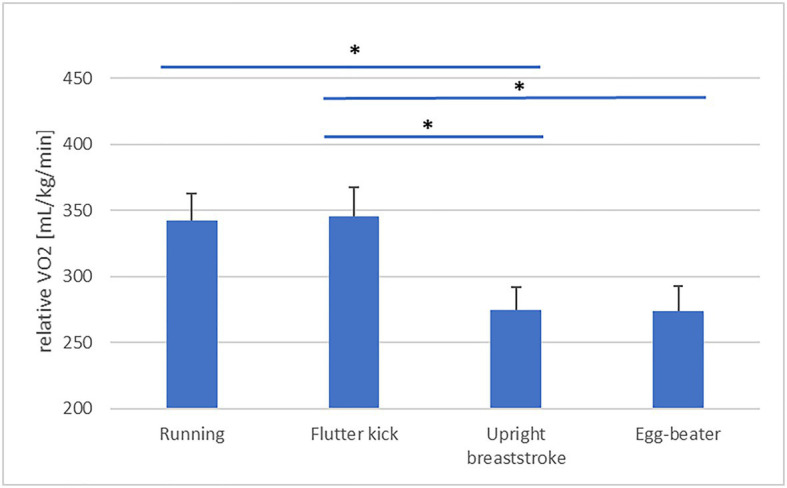
Oxygen Consumption (VO_2_) relative to in-water weight during four treading water techniques in skilled treaders – means and SEs. ^*^Denotes significant difference between techniques (*p*_corrected_<0.05).

Descriptive statistics are presented in Table 3. Our analysis revealed a significant effect of Technique on skilled treaders’ PRT [*F*(3, 60)=6.59, *p*=0.001, partial η^2^=0.25, obs. Power=0.96, see Figure 1]. *Post-hoc* analysis is detailed in Table 4. PRT was significantly faster during “upright breast-stroke” and “egg-beater” compared to “running” and “flutter kick.” There was a significant effect of Technique on NASA TLX scores [*F*(3, 60)=27.75, *p*=0.000, partial η^2^=0.58, obs. Power=1.00, see Figure 2]. *Post-hoc* analysis showed that NASA Task Load was significantly lower during “upright breast-stroke” and “egg-beater” compared to “running” and “flutter kick.” A significant effect of Technique on RPE was found [*F*(2.13, 42.59)=41.36, *p*=0.000, partial η^2^=0.67, obs. Power=1.000, see Figure 3]. *Post-hoc* analysis showed that RPE was significantly lower during “upright breast-stroke” and “egg-beater” compared to “running” and “flutter kick.” There was a significant effect of Technique on heart rate [*F*(1.97, 39.35)=8.94, *p*=0.001, partial η^2^=0.31, obs. Power=0.96, see Figure 4]. *Post-hoc* analysis showed that Heart Rate was significantly lower during “upright breast-stroke” and “egg-beater” compared to “running” and “flutter kick.” There was also a significant effect of Technique on relative oxygen consumption [*F*(3, 60)=29.10, *p*<0.001, partial η^2^=0.59, obs. Power=1.00, see Figure 5]. *Post-hoc* analysis showed that relative oxygen consumption was significantly lower during “upright breast-stroke” and “egg-beater” compared to “running” and “flutter kick.”

**Table 3:** Descriptive statistics.

	“Running”		“Flutter kick”		“Upright breaststroke”		“Egg-beater”	
	Mean	*SD*	Mean	*SD*	Mean	*SD*	Mean	*SD*
PRT (s)	0.52	0.02	0.52	0.03	0.49	0.03	0.49	0.02
TLX (score 0–50)	31.90	2.01	28.48	2.87	16.69	1.89	14.92	1.94
RPE (score 6–20)	13.92	0.39	13.13	0.50	10.79	0.37	10.59	0.33
HR (bpm)	140.39	6.68	146.91	6.47	129.41	4.66	128.97	5.99
Wet-weight relative VO_2_ (ml/kg/min)	342.30	95.51	345.74	101.33	274.60	79.88	273.99	87.83
Dry-weight relative VO_2_ (ml/kg/min)	29.02	7.40	29.37	8.56	23.47	7.28	23.18	6.31

**Table 4:** Results of repeated measures ANOVAs.

Variable	*p*	*F*	Partial η^2^	Observed power	Post-hoc analysis (correction for multiple comparisons: Bonferroni)
PRT	0.001	6.59	0.25	0.96	1=2=3=4, 1>3, 1>4, 2>4
TLX	<0.001	27.75	0.58	1.00	1=2>3=4, 1>3, 1>4, 2>4
RPE	0.001	41.36	0.67	1.00	1=2>3=4, 1>3, 1>4, 2>4
HR	0.001	8.94	0.31	0.96	1=2>3=4, 1=3, 1=4, 2>4
Wet weight- relative VO_2_	<0.001	29.10	0.59	1.00	1=2>3=4, 1>3, 1>4, 2>4

## Discussion

For skilled water treaders, the “egg-beater” technique and the “upright breaststroke” technique were found to lead to significantly lower RPE, TLX score, oxygen consumption, heart rate, and PRT compared to the “flutter kick” and “running” technique. These findings indicate that skilled treaders require less energetic and cognitive resources to perform the “egg-beater” and “upright breaststroke” techniques compared to other common techniques.

A potential reason for the differences in physical demand (HR, VO_2_, and RPE) between techniques could be that a different physical force is generated when performing sculling movements compared to kicking and pushing movements: while pushing against the water moves the swimmer up through resistance forces, sculling movements reputedly generate lift force (Schnitzler et al., 2014). In a fluid medium such as water, which is displaced when direct forces are applied, insufficient resistance is available for pushing movements to be effective. Sanders (1999) found that elite water polo players used more sculling movement and more effective ankle movement compared to less skilled players, and suggested that using a sculling type movement while minimizing the downward movement may maximize performance and conserve energy (Sanders, 1999). Furthermore, the consistency of the lift generated by continuous sculling movements, such as the “egg-beater” kick, is greater, as the movement cycle does not contain a recovery phase. A more sensitive analysis of the lift forces produced by both the feet and hands in the different water-treading techniques would be required to confirm this suggestion (Schnitzler et al., 2021). A fact that weakens this interpretation, however, is that “upright breaststroke” seems to be as good as “egg-beater” in energetic requirements – even though it does contain such a recovery phase. It is possible, in our opinion, that the combination of synchronous, lateral kicks with sculling hand movements in “upright breaststroke” was sufficiently economic for this task because participants’ hands were not otherwise occupied (and could therefore compensate for the recovery phase of the legs). If participants had to lift their hands out of water (i.e., to grab something or signal for help), we suggest that “egg-beater” would be the best solution to stay level. However, we do not have any biomechanical data to support this potential explanation. A study involving a secondary task performed by the hands while treading water with either technique, for example, would help ascertain whether this is the case.

Skilled water treaders also showed significantly faster PRTs when performing “egg-beater” kick or “upright breaststroke” technique compared to flutter kick and “running” technique, and reported a lower TLX score in these techniques. Collectively, these findings indicate that cognitive demands related to these two movement patterns may be lower compared to other treading techniques, at least in experienced water treaders. Processing of movement-related information requires the use of working memory, which may limit the capacity available to deal with other cognitive challenges, such as evaluating options and making decisions (Masters and Maxwell, 2004; Baddeley, 2012). In an emergency situation, choosing to perform “egg-beater” or “upright breaststroke” may therefore free up cognitive resources for performing other activities, such as scanning, communication, and decision making. The finding of faster PRTs during performance of the “egg-beater” and “upright breaststroke” techniques also fits with previous research in water treading (Button et al., 2019), which showed that both these techniques were more stable in changing environments (increased cognitive demands, treading with clothing and in moving water) compared to “running” and “flutter kick.”

Each measure of economy, whether cognitive or physical, showed similar trends. This may indicate that cognitive load may be influenced not only by the information-processing demands related to movement coordination but also by the metabolic energy demands. Indeed, based on observations of increasing economy in both energetic and cognitive expenditure during motor learning, Sparrow et al. (2007) suggested that the energetics (i.e., biomechanics and metabolic energy cost) and the cognitive or attentional demands of maintaining a coordination pattern should be investigated in tandem.^4^ It is likely that metabolic and cognitive requirements represent two aspects of the same phenomenon – the energetics of motor behavior (O’Dwyer and Neilson, 2000). Potentially, by selecting a treading pattern that feels less energetically demanding, humans may be simply seeking a solution to a complex degrees of freedom problem (i.e., keeping an upright position in the water) that minimizes metabolic and cognitive energy costs (Sparrow, 1983). Further analyses are required to shed light on the relationship between cognitive and energetic demands of motor performance. Interestingly, most participants chose to perform the “eggbeater” technique when treading without instruction. This may either reflect personal preference, habit or personal conviction about the most efficient technique, or an implicit understanding of movement economy.

### Practical Implications

Energetic and cognitive demand likely affect how long a person can tread water before the onset of exhaustion or potentially hypothermia (Hayward et al., 1975b; Stallman et al., 2017), and how well they may deal with additional cognitive challenges such as making decisions (Ducharme and Lounsbury, 2007). It may make sense to choose the technique that is taught to inexperienced treaders based on evidence of cognitive and energetic requirements of the different techniques. However, it is well-established that drowning incidents are multifactorial and depending on the specific situation (e.g., cold water, moving water, clothing worn, fatigued, and in panic) different behaviors may be more adequate (Button et al., 2019).

“Egg-beater” is arguably a challenging coordination pattern for inexperienced treaders to perform effectively, and other techniques might be easier to learn initially. Given the limited amount of time that is spent on teaching treading water in standard swim lessons (Moran, 2008); a learner may not reach an advanced, automated, and efficient level at the “egg-beater” technique. Regarding instruction, we suggest that a focus on “upright breast-stroke” and “egg-beater” technique in favor of “running” and “flutter kick” technique may be more useful when teaching novices, at least when it can be certain that the learner will practice long enough to achieve a high level of competence (i.e., performs the movement correctly).

### Limitations

A potential limitation arising from testing only experienced treaders is the application of findings to the general population. However, the goal of the present study was not to provide direct recommendations regarding the ideal movement pattern during an immersion incident, instead it addressed a gap in the literature relating to the basic, underlying energetic and cognitive demands that are inherent the performance of each technique. For this purpose, it was necessary to ensure correct movement execution, which is why only well-trained persons were included. The results of this study will be helpful to direct future studies that need to be conducted before recommendations for practice, or instruction, can be made. Another argument that may arise is that water polo players may have more practice in the egg-beater and upright breaststroke techniques, and are therefore better able to coordinate their limbs to perform these techniques. However, it has been repeatedly shown that water polo players practice and use a wide range of swim strokes and treading water techniques during training and competition (e.g., Smith, 1998). Also, the movement quality ratings confirm that participants performed each of the four techniques to a high standard (Table 2). As such, we assumed that the differences in energy requirement we report were not attributable to expertise differences between the patterns. A further limitation is the external validity of the findings for open water emergencies: since testing in exhaustion trials or in cold water would take a long time and put participants at risk, the present study was conducted in a controlled lab environment with ambient temperature and a sub-maximal exercise protocol. The task that was used to investigate cognitive load associated with movement coordination was selected for its feasibility; so as not to affect movement coordination or oxygen consumption measures. As such, the measure does not represent the whole range of domains of working memory that may be employed to control movement, and therefore does not represent an absolute measure of cognitive load. Lastly, the testing was conducted in a single day, which arguably could have introduced fatigue effects. However, careful counterbalancing of the order of techniques controlled for the effects of fatigue, and furthermore, reported exertion as well as physiological indices (e.g., heart rate and oxygen consumption) show that participants were overall only exercising at a moderate level. Furthermore, the use of a flume pool in the present study precludes us from extending our findings to realistic, open water environments. As a follow-up to this study, an experiment conducted in open water would be of value to increase the external validity of our findings.

### Conclusion

The “egg-beater” technique and the “upright breaststroke” technique may incur lower cognitive and energetic demands compared to other water treading techniques when performed by skilled water treaders.

## Data Availability Statement

The datasets presented in this study can be found in online repositories. The names of the repository/repositories and accession number(s) can be found below: Open Science Framework (https://osf.io/bruxa/?view_only=ea5c8f5ee0cb44e695649e57264818e2).

## Ethics Statement

The studies involving human participants were reviewed and approved by University of Otago Human Research Ethics Committee Approval # 20/004. Written informed consent to participate in this study was provided by the participants or their legal guardian/next of kin.

## Author Contributions

All authors significantly contributed to the research process. TD contributed to study concept and design, acquisition of subjects, data collection, analysis and interpretation of data, and preparation of manuscript. CB and RM contributed to study concept and design, analysis and interpretation of data, and preparation of manuscript. All authors contributed to the article and approved the submitted version.

## Conflict of Interest

The authors declare that the research was conducted in the absence of any commercial or financial relationships that could be construed as a potential conflict of interest.

## Publisher’s Note

All claims expressed in this article are solely those of the authors and do not necessarily represent those of their affiliated organizations, or those of the publisher, the editors and the reviewers. Any product that may be evaluated in this article, or claim that may be made by its manufacturer, is not guaranteed or endorsed by the publisher.

## Footnotes

^1^The HELP involves hugging one’s own knees while floating with the help of a life vest. Note that in the reported study, the emphasis was on rate of cooling; there were no comparisons made of metabolic or cognitive demands of the different treading behaviours.

^2^This never took longer than a minute.

^3^Since wet weight (with head out of the water) is much lower compared to regular body weight measured out of the water, the relative O_2_ values in the present study cannot be directly compared with relative O_2_ consumption values reported elsewhere. We have reported the VO_2_ values standardized by dry weight as well as wet weight in Table 2 to allow more intuitive understanding of the data, however, only wet weight-standardized data were used for analyses.

^4^However, note a conflicting study in water polo: in skilled athletes, performance at a decision-making task improved under very high levels of physical exertion (Royal et al., 2006).
